# EGF receptor ligands: recent advances

**DOI:** 10.12688/f1000research.9025.1

**Published:** 2016-09-08

**Authors:** Bhuminder Singh, Graham Carpenter, Robert J. Coffey

**Affiliations:** 1 Department of Medicine, Vanderbilt University Medical Center, Nashville, TN, 37232, USA; 2 Department of Biochemistry, Vanderbilt University Medical Center, Nashville, TN, 37232, USA; 3 Department of Cell and Developmental Biology, Vanderbilt University Medical Center, Nashville, TN, 37232, USA; 4 Veterans Health Administration, Tennessee Valley Healthcare System, Nashville, TN, 37212, USA

**Keywords:** epidermal growth factor, EGFR, Amphireguin, Transforming growth factor-alpha, Epiregulin, Betacellulin, HBEGF, epigen

## Abstract

Seven ligands bind to and activate the mammalian epidermal growth factor (EGF) receptor (EGFR/ERBB1/HER1): EGF, transforming growth factor-alpha (TGFA), heparin-binding EGF-like growth factor (HBEGF), betacellulin (BTC), amphiregulin (AREG), epiregulin (EREG), and epigen (EPGN). Of these, EGF, TGFA, HBEGF, and BTC are thought to be high-affinity ligands, whereas AREG, EREG, and EPGN constitute low-affinity ligands. This focused review is meant to highlight recent studies related to actions of the individual EGFR ligands, the interesting biology that has been uncovered, and relevant advances related to ligand interactions with the EGFR.

## Introduction

Although the role of the epidermal growth factor receptor (EGFR) in the generation of biological responses has been reviewed extensively, an analysis of EGFR ligands, crucial initiators of these responses, has not been reviewed recently ^1– 8^. All EGFR ligands are synthesized as type 1 transmembrane precursors that undergo extracellular domain cleavage to release soluble ligands, which then bind to and activate the EGFR. This cleavage event is usually mediated by members of the a disintegrin and metalloprotease (ADAM) family. Understanding how these ligands are trafficked within the cell and released at the cell surface has the potential to produce significant new insights in cell biology. For example, the role of a trafficking adaptor for the transforming growth factor-alpha (TGFA) precursor (see ‘Transforming growth factor-alpha’ section) and the role of exosomal ligands in mediating receptor activation (see ‘Amphiregulin’ section) have been identified during these studies.

## Aspects of individual ligands

In the following paragraphs, recent advances for each of the seven EGFR ligands are discussed. Although in this section we discuss the ligands individually, we point out that these ligands do not act in isolation but rather affect the behavior of each other to accomplish a diverse repertoire of biological responses through EGFR signaling. From a focused view in this section, the next section highlights the differences, similarities, and cross-talk among the ligands.

### Epidermal growth factor

EGF is the prototypic and founding member of the EGFR ligand family, first identified from submaxillary gland extracts during nerve growth factor studies ^9^. The EGF-EGFR ligand-receptor system has greatly enhanced our understanding of receptor tyrosine kinase signaling, as evidenced by more than 70,000 publications for EGF alone. A recent review has distilled our current understanding of EGF and its actions ^3^. More recently, a study uncovered that EGF-induced EGFR signaling enhances production of intracellular reactive oxygen species (ROS) by dual oxidase 1 (DUOX1) ^10^. This nicely complements earlier studies in which ROS were shown to enhance EGFR signaling by modulating both positive and negative regulators of EGFR signaling (ADAMs and protein tyrosine phosphatases) ^11– 14^. In another recent study, urinary EGF has been shown to be an independent risk factor for progression of chronic kidney disease, substantiating earlier findings by Harris and colleagues ^15– 18^.

### Transforming growth factor-alpha

A historical perspective of key advances for TGFA, including TGFA regulation at the level of expression, trafficking, and processing, has been provided in a recent review ^7^. Studies with transmembrane TGFA precursor (pro-TGFA) uncovered a novel interaction with Naked2 (NKD2) and showed that NKD2 acts as a cargo recognition and targeting (CaRT) protein for pro-TGFA ^19, 20^. In polarized epithelial cells, NKD2 envelops pro-TGFA-containing exocytic vesicles and directs them to the basolateral surface where the vesicles dock and fuse in an NKD2 myristoylation-dependent manner ^19^. In Madin-Darby canine kidney (MDCK) cells expressing myristoylation-deficient NKD2 (glycine at the second position is replaced by an alanine, G2A-NKD2), the vesicles accumulate at the basolateral “corner” and pro-TGFA is trapped in the cytoplasm ^20^. Basolateral delivery of pro-AREG and pro-EREG, unlike pro-TGFA, is unaffected in G2A-NKD2-expressing MDCK cells, suggesting utilization of alternate trafficking machinery ^21, 22^. More recently, the Schekman lab has demonstrated that cornichon-1 (CNIH) acts as a cargo receptor for pro-TGFA in the early secretory pathway ^23^. These findings indicate that each ligand has distinct nuances as to its biosynthetic trafficking, cell surface delivery, and ectodomain cleavage. Upstream of regulation at the protein trafficking level, TGFA can be regulated at the level of translation by microRNAs (miRs) directly or indirectly (for example, by miR-374a ^24^ and miR-505 ^25^ directly and by miR-124 through Slug ^26^, in addition to other miRs reported earlier ^7, 27^). miR-203 has been noted to be a broad EGF family regulator that binds to 3′ untranslated regions of AREG, EREG, and TGFA mRNA and regulates their stability ^28^.

### Amphiregulin

**Figure 1.**
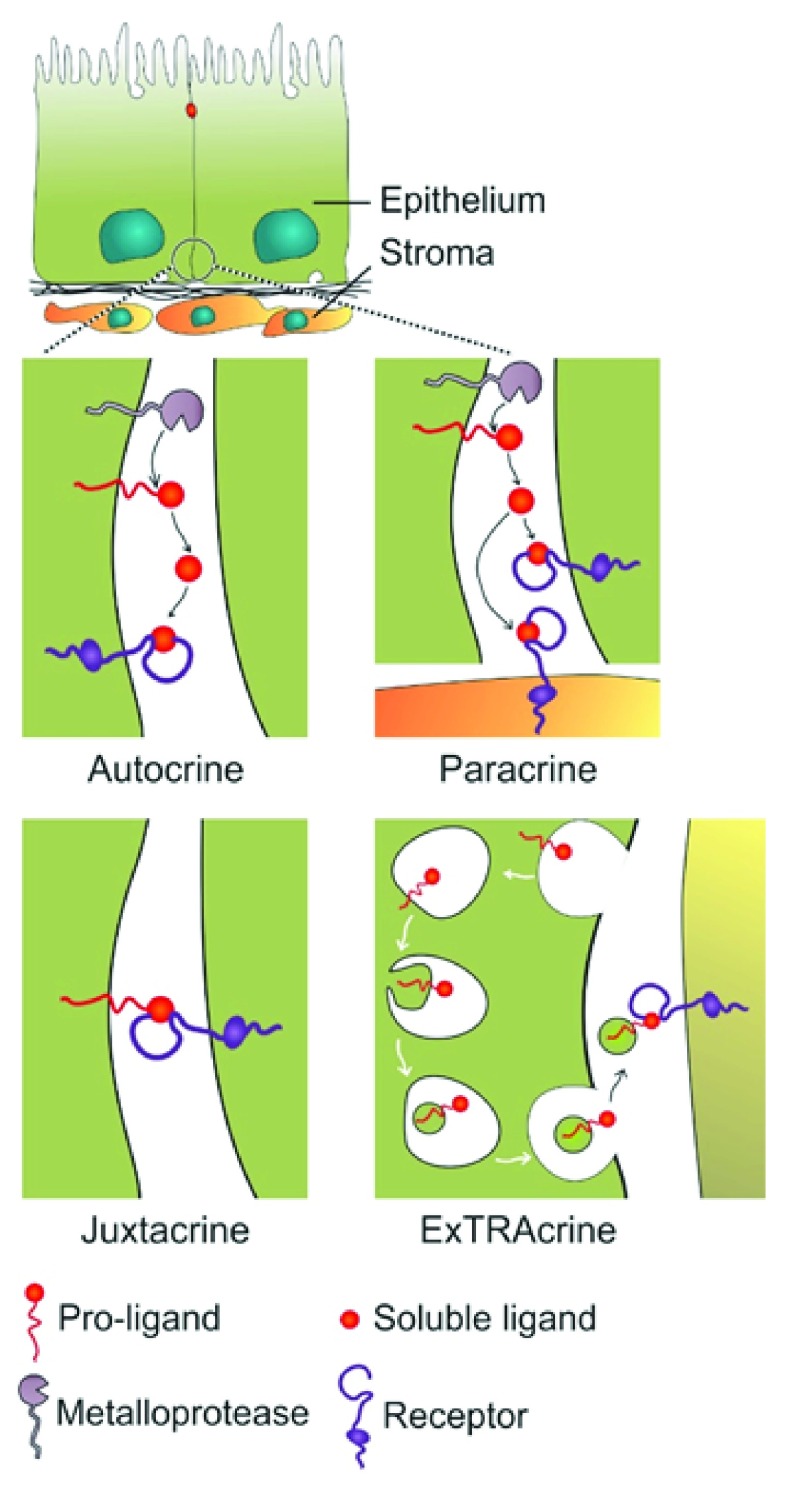
Modes of signaling via epidermal growth factor receptor (EGFR) ligands. Autocrine signaling occurs when a ligand is released from a cell and binds to EGFR on that same cell. Paracrine signaling refers to the released ligand acting on a nearby cell, usually a different cell type. Juxtacrine signaling occurs when a non-cleaved, transmembrane ligand binds to EGFR on an adjacent cell; this is best documented for heparin-binding epidermal growth factor-like growth factor (HBEGF). Amphiregulin (AREG), transforming growth factor-alpha (TGFA), and HBEGF, as well as EGFR, can be packaged into signaling competent exosomes. Uptake of exosomal AREG by recipient cells is, at least in part, dependent on EGFR, leading to the term exosomal targeted receptor activation (ExTRAcrine). ExTRAcrine signaling has features of autocrine, paracrine, and juxtacrine signaling as well as possibly endocrine signaling since EGFR and AREG can be detected in human plasma exosomes ^30^. Adapted from Singh and Coffey ^36^.

A recent review has highlighted our current understanding of AREG ^2^. During pro-AREG studies, a new mode of EGFR ligand signaling via exosomes was discovered ^29^. pro-AREG is packaged into exosomes, and pro-AREG-containing exosomes increase the invasiveness of recipient breast cancer cells. Exosomal uptake is partially dependent on ligand-receptor interaction as treatment of recipient cells with EGFR blocking monoclonal antibody attenuated the uptake of pro-AREG-containing exosomes. We have termed this new mode of EGFR activation by exosomal ligands as ExTRAcrine (exosomal targeted receptor activation) signaling. As noted in Figure 1, ExTRAcrine signaling has features of autocrine, paracrine, and juxtacrine signaling. It is possibly involved in endocrine signaling as well, since EGFR and pro-AREG can be detected in human plasma exosomes ^30^.

There is accumulating evidence that AREG is produced in a number of cells other than epithelial cells and fibroblasts. Artis and colleagues have identified roles for AREG in immune surveillance ^31^. Damaged epithelial cells release interleukin-33 (IL-33), IL-25, and thymic stromal lymphopoietin (TSLP), which can activate group 2 innate lymphoid cells to release AREG, as well as IL-5, IL-9, and IL-13. It is thought that AREG ameliorates injury by binding to epithelial EGFR and stimulating proliferation and repair. This does not exclude an effect of AREG on EGFR-expressing, non-epithelial cells, including fibroblasts, polymorphonuclear cells, and Fox3 ^+^ regulatory T cells; the latter two have been reported to express EGFR by flow cytometry ^31, 32^. A difficulty in studying the actions of the ligands in the mouse is the lack of robust antibodies to examine mouse EGFR by immunofluorescence or immunohistochemistry, or both.

A variety of stresses, such as inflammation (with lipopolysaccharides), ischemia, and hypoxia, induce AREG and EREG expression in the brain (cortex, striatum, and hippocampus). Under these stresses, glial cells show upregulation of EREG and AREG, which, when released, may protect against neuronal cell death. In Neuro2a cells, administration of EREG or AREG inhibits tunicamycin-induced endoplasmic reticulum (ER) stress and cell death ^33^. Recent work by Elder’s group continues to implicate a role for the C-terminal domain of AREG in promoting keratinocyte proliferation ^34^. In addition, Yarden’s group has shown preclinical efficacy for an AREG neutralizing antibody in ovarian cancer ^35^.

### Epiregulin

EREG binds to and activates EGFR and ERBB4. EREG is weakly expressed in normal adult tissues but is overexpressed in diseases like cancer. Multiple roles for EREG in normal physiology and disease states have been reviewed recently ^4^. However, the basic biological processes, such as the polarized distribution of pro-EREG within cells and the subsequent spatial control of EGFR activity, have been understudied. We have identified a new mode of epithelial transformation by apical mistrafficking of pro-EREG ^22^. In polarized MDCK cells, pro-EREG is delivered to the basolateral membrane and its delivery is dependent on a tyrosine-based (YXXΦ) sorting motif. Disruption of this motif leads to complete apical delivery; however, pro-EREG basolateral sorting is independent of the usual YXXΦ-recognizing clathrin adaptor, AP1B ^22^. This apical rerouting of pro-EREG leads to activation of apical EGFR signaling, which has a different activation profile compared with basolateral EGFR signaling. MDCK cells expressing an apical mistrafficking mutant of EREG (Y156A) are more transforming than their wild-type, basolateral EREG-expressing counterparts when injected subcutaneously in nude mice. Additionally, there are mutations in human cancers that would disrupt the sorting motifs of the majority of EGFR ligands, including pro-EREG ^36^. In a recent study with breast epithelial MCF10A cells, EREG expression was shown to contribute to tumor progression during early stages of cancer ^37^. A comprehensive understanding of pathways that govern spatial compartmentalization of the EGFR ligands might reveal alternate approaches to treat cancer.

Recently, additional functions of EREG have been uncovered. In circulating monocytes, EREG is upregulated acutely during short bursts of exercise and intermittent hypoxia in rat aortic smooth muscle cells. These facts may have implications for atherosclerosis ^38^. Additionally, EREG is shown to assist in the proliferation, repair, or regeneration (or a combination of these) in liver, colon, and salivary gland acinar cells ^39– 42^. EREG also plays a role in odontogenesis by enhancing proliferation of dental apical papilla stem cells and inducing oral epithelial cell differentiation by dental papilla cells ^43^.

Wrana and colleagues have identified EGFR-dependent Yap signaling in the intestine which contributes to regeneration and tumorigenesis ^44^. They reported that EREG, but not EGF or AREG, is able to maintain growth of organoids generated from Yap null mice; however, it should be noted that the concentration of recombinant mouse EREG given was very high and the source of the other ligands was unclear.

### Betacellulin

Betacellulin (BTC) is a dual-specificity ligand that binds to and activates EGFR and ERBB4. In a recent review, structural details and key functions of BTC gleaned from knockouts, transgenic animals, patient samples, and *in vitro* studies have been reviewed ^8^. Recently, it has also been shown that pro-BTC localizes to the basolateral membrane ^45^. pro-BTC basolateral sorting is dependent on a cytoplasmic EEXXXL motif, and disruption of this motif, or introduction of a human cancer mutation (E156K) within this motif, leads to pro-BTC mistrafficking. An analogous EEXXXL motif is also responsible for basolateral sorting of pro-AREG ^21^. This report also demonstrated that pro-BTC mistrafficking induces an EGFR-dependent hepatic polarity phenotype (apical surfaces on the side, between two cells) in otherwise columnarly polarized MDCK cells, a finding not observed with any of the other EGFR ligands.

Recent publications have uncovered additional functions of BTC. BTC transgenic mice display high cortical bone mass ^46^. Additionally, in bone metastases associated with castration-resistant prostate cancer, BTC is upregulated in osteoblasts and contributes to osteoblastic activity ^47^. BTC transgenic mice also develop urothelial hyperplasia and show sex-dependent reduction in urinary protein content, which appears to be independent of EGFR signaling, suggesting a role for ERBB4 ^48^. BTC has also been identified as a novel modulator of interferon (IFN) response and enhances the anti-viral action of IFN ^49^. In a large cytokine and chemokine screen to modulate IFN responses, BTC was identified as one of the most potent modulators of the IFN response. Moreover, miR-200 has been shown to control BTC (and AREG) translation ^50^.

### Heparin-binding epidermal growth factor-like growth factor

Roles for heparin-binding EGF-like growth factor (HBEGF) in multiple cellular processes in normal and disease states have been recently reviewed ^5^. Among the EGFR ligands, pro-HBEGF has the longest residency time at the cell surface, perhaps explaining why pro-HBEGF functions as a receptor for the B fragment of Diphtheria toxin. Since the toxin binds only to human and monkey pro-HBEGF, mouse modelers have exploited this selective binding by introducing human pro-HBEGF into the ATG start site of mouse genes expressed in selected cell types, which can then be eliminated by administration of the toxin ^38, 39^. However, when repurposing pro-HBEGF in this way, possible “side effects” of human HBEGF (that can bind to mouse EGFR and ERBB4) need to be considered ^51^.

Local administration of HBEGF helps mice recover from chronic suppurative otitis media, a chronic inflammation of the middle ear ^52^. Delivery of EGF or FGF2 was not effective ^53^. It is unclear whether auto- or cross-induction (or both) of other ligands play a role in this process ^54, 55^. HBEGF mRNA is also a target for miR-132, both of which play a major role in wound healing ^56^. During the transition from inflammation to proliferation in wound healing, miR-132 expression was upregulated together with a concomitant decrease in HBEGF levels. Surprisingly, HBEGF downregulation coincided with the proliferative phase during wound healing and increased receptor activity. Other miRs (for example, miR-96, miR-212, and miR1192) have also been shown to target HBEGF ^57– 59^.

### Epigen

Epigen (EPGN), the most recently discovered EGFR ligand, seems to be a low-affinity EGFR ligand. The localization of pro-EPGN in polarized epithelial cells is not known and its cytoplasmic domain lacks any recognized basolateral sorting motifs ^36^. Schneider and Yarden have recently reviewed EPGN structure and function ^6^. EPGN knockout mice do not display an obvious phenotype ^60^; however, transgenic overexpression of EPGN during embryonic development induces sebaceous gland hyperplasia ^61^. Interestingly, activation of the transcription factor Nrf2, a master regulator of cellular anti-oxidant defense, causes sebaceous gland enlargement in an EPGN-dependent manner ^62^. Pharmacologic activation of Nrf2 has been employed as a cancer prevention strategy and this is due in part to its role in ROS detoxification ^63^. However, Nrf2 activation-induced EPGN upregulation and subsequent EGFR activation might actually be pro-tumorigenic and act counter to its anti-cancer effects. EGFR signaling also regulates Nrf2 activity; in cortical neurons, astragaloside IV (extracted from *Astragalus membranaceus*) induces HBEGF-dependent EGFR transactivation that leads to Ser40 phosphorylation of Nrf2 and its nuclear translocation ^64^. EPGN transgenic mice also show a peripheral demyelinating neuropathy, leading to late-onset muscular dystrophy ^65^.

## Different ligands, differing functions

EGFR can be activated by seven related, but distinct, ligands. Multiple publications have noted the capacity of these different ligands to act in a “functionally selective” manner (that is, to produce quantitatively and, to a lesser extent, qualitatively distinct cellular responses ^66– 68^). It is important to note that EGFR is one of four members of a family of related receptors. The others are designated as ERBB2/HER2, ERBB3/HER3, and ERBB4/HER4. Of the ligands that bind to the EGFR, three (EREG, HBEGF, and BTC) are known to bind to and activate ERBB4. None of the EGFR ligands is known to interact with ERBB2 or ERBB3. A family of EGF-related ligands, termed neuregulins, binds to ERBB3 and ERBB4. There is no known ligand for ERBB2.

In some cases, the ligand functional selectivity could be due to the capacity of some of the ligands to activate ERBB4; however, differences also exist in cells that do not express detectable levels of ERBB4. As noted above, four ligands (EGF, TGFA, AREG, and EPGN) interact solely through EGFR, yet they do not produce identical biological responses. In particular, AREG is often regarded as a low-affinity ligand for the EGFR. Crystallographic studies have described high-resolution ligand:ectodomain structures for EGF:EGFR, TGFA:EGFR, and NRG-1β:ERBB4 ^69^. However, these studies do not provide an explanation for downstream differences in biological activity.

Recently, a comparative study ^70^ of EGF, TGFA, AREG, and BTC binding to the EGFR and subsequent dimer formation has made clear that EGF and TGFA have a distinct preference to produce EGFR:ERBB2 heterodimers compared with EGFR:EGFR homodimers, but that BTC and AREG produced dimers of both types equally. In addition, AREG produced significantly (50%) fewer dimers of either type compared with the other ligands. These initial data point to dimerization-competent, conformation-based receptor differences provoked by different ligands as a potential basis for heterogeneity in signaling and biological outcomes. These data and the related issues of receptor methylation and receptor antagonists (discussed below) will need to be evaluated.

Another fact, sometimes forgotten or ignored, is that EGFR ligands can auto- and cross-induce one another, adding a layer of complexity to studies of an individual ligand ^54, 55^. In addition, the often observed co-expression of EPGN, EREG, AREG, and BTC, which may be due to their chromosomal clustering on human 4q13-21 and 5E1 in the mouse, further complicates our understanding of the role of individual ligands in biological processes ^55^. ExTRAcrine signaling and auto- and cross-induction of EGFR ligands are variables that merit consideration in systems biology approaches to the merging fields of autocrine signaling and quorum sensing, as reviewed elsewhere ^71^.

Interestingly, exogenous administration of ligands has recently been used as a treatment strategy for certain disease conditions in mice. Table 1 lists distinct actions recently observed for individual EGFR ligands *in vivo*. Once again, this is a highly selective compilation and we apologize for any omissions. We have chosen to highlight studies that identify new functions of the individual ligands.

**Table 1.** *In vivo* administration of epidermal growth factor receptor ligands as treatment strategies.

Ligand	Mode of administration	Effect	Reference
EGF	One-week perfusion with ciliary neurotrophic factor via mini-osmotic pump	Acinar to beta cell transdifferentiation for up to 248 days in adult mice with chronic hyperglycemia	88
HBEGF	Intranasal	Reduced oligodendrocytic death when given immediately after injury in a mouse model of pre-term brain injury	89
HBEGF	HBEGF-containing hydrogel injected through the external auditory canal	Regeneration of chronic tympanic membrane perforations in mice	52
HBEGF	Topical application	Accelerated wound healing in a diabetic mouse model	90

Exogenous administration of the soluble epidermal growth factor receptor ligands for the treatment of various disease states in animal disease models. EGF, epidermal growth factor; HBEGF, heparin-binding epidermal growth factor-like growth factor.

## Ligand processing and delivery to the cell surface

As noted above, all seven mammalian EGFR ligands are synthesized as a type 1 transmembrane precursor and are processed through biosynthetic compartments common to other secretory and cell surface proteins. We have mentioned earlier that mammalian ligands are usually cleaved by ADAMs. Owing to the focus on EGFR ligands in this review, we have not elaborated on ADAM activity in cleavage of EGFR itself. ADAM-mediated EGFR cleavage acts as a negative feedback for EGFR signaling and acts in concert with the positive feedback through ligand shedding ^72^. *Drosophila* genetics has identified the involvement of particular gene products (Rhomboid, Star) for intracellular trafficking of the fly EGFR ligands. Recent work in mammalian cells has found at least two novel roles for inactive rhomboids (iRhoms) in the processing of EGFR ligand precursors ^73^.

Rhomboid gene products are known to be seven membrane-spanning molecules, which function as intramembrane serine proteases that cleave various transmembrane molecules within the cell or at the cell surface ^73^. The rhomboid family also includes catalytically inactive proteins termed iRhoms. Although rhomboid cleavage of the fly EGFR ligand precursor is required for processing to the cell surface, the presence of iRhoms in the biosynthetic pathway prevents this cleavage and leads to intracellular degradation of EGFR ligand precursors ^74^. iRhoms localize to the ER. In this way, iRhoms block the cell surface expression of multiple EGFR ligands, several of which are not substrates for the catalytic rhomboids. Hence, the iRhoms may not just compete with rhomboids, but rather regulate EGFR ligand levels by an independent mechanism, suggesting that the iRhom gene may have evolved a distinct regulatory function. Consistent with this idea, during evolution the iRhoms have acquired large segments of sequence not shared with the catalytic rhomboids. In flies, the independent mechanism that regulates intracellular ligand levels is identified as the ER-associated protein degradation pathway that leads to proteosomal degradation ^73^.

A second, less direct, mechanism by which iRhoms control the production of bioactive secreted EGFR ligands is through control of the ultimate step in processing: the metalloprotease-mediated, cell-surface cleavage of the ligand precursor. This step is executed by members of the ADAM protease family of which ADAM17 is probably the most significant member for EGFR ligands. ADAMs are single-pass transmembrane proteins that are trafficked through the ER and Golgi before reaching the cell surface, where the ectodomain proteolytically cleaves the ectodomain of substrates, such as EGFR ligand precursors. During intracellular trafficking, ADAMs are converted by furin-dependent proteolytic processing in the Golgi from an inactive form to a mature active species, and this requires iRhoms ^75– 78^. When mammalian iRhoms are deleted or knocked down, no mature cell surface ADAM17 molecules are produced and in turn ADAM17 substrates, such as EGFR ligand precursors are not cleaved. In mammals, there are two iRhoms (RHBDF1/iRhom1 and RHBDF2/iRhom2), which seem to have overlapping effects on ADAM17 maturation, depending on the cell type. iRhom effects, however, are selective toward ADAM17. Since ADAM17 also cleaves the inflammatory tumor necrosis factor precursor, iRhoms may influence both cell proliferation and inflammatory pathways. Interestingly, dominant iRhom2 mutations have been detected in an inherited syndrome, tylosis (thickening of the palms and soles), in patients with esophageal cancer ^79^. The disorder appears to be due to mutations in the N-terminal cytosolic domain of iRhom2 that stabilize the protein. These mutations have been linked to increased release of AREG (and HBEGF) and increased EGFR activity. The impact of these mutations on ADAM17 activity is unsettled ^79– 81^. A spontaneous recessive mutation in this domain of iRhom2 has been identified in a mouse with a hairless phenotype called curly bare (cub) ^82^. Of interest, there is a suppressor of Cub, Mcub, in which there is a loss-of-function mutation in mouse AREG ^82^.

## Ligand interactions with receptors

Although the complexities presented by multiple ligands binding to multiple ErbB receptors are described above and in more detail elsewhere ^68, 70^, a few recent publications give additional parameters to consider. First, an antagonist has now been described for the mammalian receptors ^83^, which adds to the negative control of ligand receptor interaction described some time ago for the *Drosophila* EGFR system. In flies, the secreted molecule Argos is able to associate with fly EGF and prevent ligand binding to the *Drosophila* EGF receptor (DER) ^84, 85^. Although an Argos equivalent has not been detected in mammals, Zheng and colleagues ^83^ report that migration inhibitory factor (MIF), which is O-glycosylated and secreted, binds to EGFR and blocks EGF binding. This antagonist is, therefore, mechanistically distinct from Argos. The report does show that MIF blocks EGF binding to its receptor in cell culture, preventing activation of the receptor and downstream signaling pathways, and that recombinant MIF interacts with the recombinant EGFR ectodomain in a purified system, but there are a few missing pieces to this provocative and potentially significant report. Does MIF interact with the EGFR at biologically significant concentrations? What is the receptor interaction site for MIF and does it overlap with the known ligand binding sites? Is there specificity to the MIF interaction within the ERBB system or unrelated receptors? Importantly, this study also shows that EGF activation of its receptor induces the secretion of a metalloprotease (MMP 13) that degrades MIF and thus provides a feedback loop to this EGFR antagonist.

The EGFR is subject to a variety of co- and post-translational modifications that have significant roles in the capacity of the receptor to transduce second messenger systems following ligand binding. Recently, the role of methylation in mediating high-affinity ligand binding has been described ^86^. Methylation at R198 and R200 within the EGFR ectodomain is reported to mediate high-affinity EGF binding. When receptor methylation is prevented by mutagenesis of the two Arg residues or by knockdown of the relevant methyltransferase (PRMT1), dissociation constant (Kd) values are altered approximately threefold to reflect a loss of higher affinity binding compared with the wild-type receptor. Decreased ligand-dependent receptor dimerization, activation, and downstream signaling, including tumorigenesis, are observed in the absence of receptor methylation, compared with wild-type receptor. Similarly, exogenous expression of PRMT1 increased high-affinity ligand binding to the EGFR, as well as receptor-mediated downstream signaling events. The study also provides evidence that a pool of PRMT1 is localized within the ER.

The EGFR ectodomain is often subdivided into four regions (D1, D2, D3, and D4) with EGF binding requiring molecular contacts with D1 and D3. In untreated cells, over 90% of the receptor exists in an inactive or “tethered” state involving interaction of residues in D2 and D4 that sterically prevent ligand binding to the D1 and D3 regions. Methylation of R198 and R200, located in D2, is proposed on the basis of molecular modeling to destabilize the tethered conformation and thereby increase receptor in the extended conformation, which allows high-affinity binding to D1 and D3. In the past, the basis of high-affinity binding has been attributed to receptor heterogeneity or negative cooperativity ^87^. Recent studies that have supported these mechanisms may have to be adjusted to include receptor methylation status. The level of methylated receptor is estimated to be approximately 10% of the EGFR population, which is about the same as the high-affinity binding receptor pool. Since methylation of the EGFR ectodomain is reported to occur within the lumen of the ER/Golgi during biosynthesis, the authors concluded that the level of PRMT1 activity in that compartment may determine the size of the high-affinity receptor pool. If the above studies are confirmed, methylation may also contribute to high-affinity binding.

Analyses of colorectal tumor tissue showed an increased level of methylated EGFR compared with control tissue. Methylated receptor was correlated with a worse overall patient survival and higher recurrence rate. Cetuximab, an EGFR neutralizing monoclonal antibody, is used to treat certain cancers, particularly colon cancer. This antibody binds to the D3 region of the EGFR, thereby blocking ligand binding. Methylation not only increased high-affinity EGF binding but also decreased the capacity of cetuximab to interfere with ligand binding. In tissues of patients with cancer, the presence of methylated receptor was a predictor of poor patient response to this therapeutic agent.

In summary, study of the EGFR and its ligands, since their discovery more than fifty years ago, continues to yield important insights into multiple biological processes. From oocyte maturation, blastocyst implantation, and embryonic development to organ development and maintenance and diseases like cancer, this line of investigation continues to be broadly relevant and clinically important.

## Abbreviations

ADAM, a disintegrin and metalloprotease; AREG, amphiregulin; BTC, betacellulin; EGF, epidermal growth factor; EGFR, epidermal growth factor receptor; EPGN, epigen; ER, endoplasmic reticulum; EREG, epiregulin; ExTRAcrine, exosomal targeted receptor activation; HBEGF, heparin-binding epidermal growth factor-like growth factor; IFN, interferon; IL, interleukin; iRhom, inactive rhomboid; MDCK, Madin-Darby canine kidney; MIF, migration inhibitory factor; miR, microRNA; NKD2, Naked2; TGFA, transforming growth factor-alpha.
